# A new study of dynamic mechanical analysis and the microstructure of polyurethane foams filled

**DOI:** 10.55730/1300-0527.3371

**Published:** 2022-02-23

**Authors:** Noureddine BOUMDOUHA, Zitouni SAFIDINE, Achraf BOUDIAF

**Affiliations:** 1 Laboratoire Génie des Matériaux, Ecole Militaire Polytechnique, Bordj El-Bahri, Algeria; 2 Laboratoire de Chimie Macromoléculaire, Ecole militaire Polytechnique, Bordj El-Bahri, Algeria

**Keywords:** Polyurethane foams, compression tests, dynamic mechanical analysis, differential scanning calorimetry, thermogravimetric analysis, scanning electron microscopy

## Abstract

Polyurethane foams have good shock-absorbing properties. This article discusses the study of the physical, dynamic analysis, and microstructure of filled polyurethane foams (PUR). We used mineral fillings nanoparticles of titanium dioxide (TiO_2_) and calcium carbonate (C1) to support and strengthen the foam cell structure to develop shock absorption and thermal resistance properties. Dynamic mechanical analysis (DMA) and compression tests compared the mechanic characterization results with different modelling approaches. For studies of physicochemical properties, we used differential scanning calorimetry (DSC) and thermogravimetric analysis (TGA). We deduced the flame retardancy mechanism. It appears that a detailed description of the characteristics of viscosity and yield stress must take into consideration the filler’s size in comparison to the cell wall’s size. The effect of size distribution on the foam’s microstructure was given by scanning electron microscopy (SEM). Half-open spherical cells were shown to be reduced in size with filling. The filler diffusion in polyurethane foams was used to model the composite foam. We observed that crystalline filler particles were uniformly distributed in the matrix, indicating that the total size is related to the density and is a crucial metric for the level of reinforcement.

## 1. Introduction

Both scientific and corporate communities have long been interested in high-performance polymer research [1]. The flexibility of polyurethane foam is ideal for mechanical and dynamic properties such as vibration and damping [2]. Due to the cell structure that distinguishes it is excellent for thermal and acoustic insulation properties and resistance to fungi, mould, and many chemicals, it is used in automobiles, electronics, industrial and medical construction, orthopaedic technology [3], and many other sectors. Due to the fundamental distinctions between gases plus solids, foams are one-of-a-kind mixtures with unique application features. For example, because polyurethane foam has a cell structure that can control flow velocity, disperse disturbance, and increase mass transfer area, it has been created for various innovative uses to enhance our lives or investigate enigmatic cosmos.

Foams expand during polymerization by producing gaseous vapours in concert with polymerization. A frequently used method of generating gas has been the chemical reaction between isocyanates and water to form carbon dioxide. Additionally, exothermic polymerization reactions can provide heat to vaporize volatile blowing agents such as dichloromethane DCM. These gases include voids that fill the area of polyurethane foam. Porous features inside the polyurethane foams are gaseous gaps surrounded by a dense phase or solid-gas composite. Under elevated pressure, the polyurethane foams get saturated by carbon dioxide CO_2_. The resulting gas bubbles produce the foam’s pores as the gas is exerted.

Some polymer materials that can be utilized in various scientific and economic industries are polyethylene and polypropylene. In all industrial applications, polyurethane foam (PUR) has been used as a shock-absorbing and combustion-resistant insulating material. It has recently been noteworthy for its mechanical characteristics that have drawn many researchers to polymers [4]. The efficient use of technology to create these materials by adding reinforcing elements as a host filler has spurred the discovery of innovative materials and techniques of installation, such as spraying linings. Polyurethane foam is made by mixing polyisocyanates with multifunctional hydroxyl groups. The urethane or polyurethane resin system represents many polymers, including urethane chemical classes, but is connected to isocyanates’ chemistry [5].

Polyurethane foam PUR is widely utilized in many applications due to its benefits in terms of energy absorption, thermal qualities, and particular strength [6,7]. Closed-cell foams are composed of a gas phase derived from various physical and chemical causes and a solid phase where the foam’s structure is produced. Foams’ polyurethane research stems from the desire to modify their physical and mechanical characteristics. One of the main facets of change in the processing of polyurethane products is the exclusion of hazardous substances from the production method and their substitution with environmentally safe ones [8]. As a result, including a closed or open cell skeleton is more regular [9]. In addition, polymer foams exhibit several fascinating mechanical features, including a large capacity for energy absorption, which is particularly important for shock dampening [10]. The mechanical characteristics of these cells are determined by the inherent factors and architecture of the polymers used to construct the cell wall structure. Foaming morphology includes cell wall thickness, average diameter, and cell shape.

The polymer’s intrinsic properties also determine the development for which a specific polyurethane foam is suitable. For example, foams derived from polyurethane with a temperature glass transition Tg close to ambient will demonstrate greater flexibility and delicacy in their material properties than foams derived from polyurethane with such a high-temperature Tg [11]. Foam characteristics are also dependent on the form and shape of the cells. For example, closed-cell foam has more heat insulation than most open-cell foams. Moreover, many physical and mechanical characteristics of foams with high aspect ratio cell structures display anisotropic behaviour [12]. Foams may also be a mixture of various solids included in the polymer matrix to alter specific physical or mechanical characteristics. Metal, glass, or ceramic powders are all such additions or fillers. These mixed foams may exhibit higher strength-to-weight ratios and energy-absorbing characteristics than foams made entirely of matrix polymers [13].

Theoretical studies on foam have been primarily focused on low-density polyurethane foams. After that, the foaming structure is represented as a compacted arrangement of cells made up of walls and struts. Gibson and Ashby’s model is the most well-known for predicting the mechanical characteristics of polymeric structures [9]. Because more excellent density foams are composed of closed and isolated spherical cells, they are classified as elastic materials. Then, utilizing the modelling produced for composite descriptions, the modelling may be executed. For example, Siegmann et al. [14] predicted that they could accurately predict the elastic characteristics of polyurethane foams using the Kerner formulas [15]. At the same time, the authors [16] modelled polymer foams with densities ranging from 0.3 to 0.86 by using procedures Christensen and Lo [17] and Herve or Zaoui [18].

The purpose of this paper is to develop and innovate shock-and flame-retardant polyurethane foams by examining the properties of stiff polyurethane foams that are strengthened with mineral particles of various sizes. Additionally, titanium dioxide nanoparticles and calcium carbonate with an intermediate particle size are employed as fillers. The SEM technique is used to characterize the architecture of polyurethane foams generated. Their mechanical properties are determined using extensive and minor deformation testing. Comparisons have been made between the experimental outcomes and several modelling schemes, such as Ashby and Gibson [9] and Christensen and Lo [17]. The modelling schemes have been updated to compensate for the effect of the filler’s size.

A variety of solvent-based polyurethanes, also poly(urethane-acrylate)s were made to compare thermal characteristics [19,20]. In the literature, studies have focused on the thermal characteristics and flame retardant properties of polyurethane foams [20]. Çakmak et al. [21] studied the effect of quantitative chain extender on some properties and performance of polyurethane for biomedical applications. Çetin [22–24] investigated the result of MWCNT particles on the polyurethanes adhesive on the low-velocity impact behaviour of sandwich panels. It was found to say that the MWCNT reinforcement of the PU adhesive significantly improved the impact resistance. The different polyurethane adhesives and honeycomb cell sizes were used to fabricate sandwich carbon fibre-reinforced composite (CFRC) structures. Gençtürk et al. [25] have also studied the filling materials of EC electrospun nanofibers into a PU foam matrix and used as a drug carrier to release a water-soluble drug sustainably. In Südemen and Önen’s study [19], phosphorus-containing polyester polyols were used to synthesize the polyurethanes and poly(urethane-acrylate)s to compare the thermal characteristics of solvent-based polyurethanes and poly(urethane-acrylate)s.

This study aims to discover how the additives affect polyurethane foam materials. This new multiapplication model will be resistant to stress and heat. We evaluated the structural polyurethane foam’s mechanical properties after applying adequate loads. When choosing a mineral filler phase, the additive’s impact on the foam’s insulating characteristics is a significant aspect to consider. Mineral particles may considerably increase polyurethane foam heat conductivity. They help encase a self-heating structure within a storage or transit container. In this scenario, modifying the foam’s dissipative properties is crucial to maintaining a safe interior temperature. The current work stems from changing the foam’s heat transmission properties. Suppose metal-laden foams are used in this application. In that case, the filling phase’s effects on material characteristics must be considered (as the foam must also defend one of the components from impacts). Polyurethane foam is loaded to determine its strength and modulus.

We have prepared and studied polyurethane foams (PUR) by sed different ratios of filling material nanoparticles of titanium dioxide (TiO_2_) and calcium carbonate (C1) to enhance good shock absorption and thermal resistance. The interaction of the viscoelastic characteristics of polyurethane foams supplemented with various ratios of fillers that may be reproduced within the range of the investigated densities. Polyurethane foam is manufactured using evaporating materials to form blanks in a polyurethane foam matrix. A sufficient amount of DCM and DBP is used to give all of the gas required to achieve the extremely low density. In that case, excessive exothermic heat is released in the thermal degradation of the developed thermoplastic polymer polymeric material to prevent overheating during polyurethane foaming. Water and isocyanate are combined to form liquid carbon dioxide, which can absorb heat and convert it to carbon dioxide gas in the manufacture of low-flexible polyurethane foam, which is resistant to heat and shock at the same time.

This paper presents a new generation of polyurethane foams with higher performance that combines very high mildness and flexibility with enhanced thermal isolation performance currently used in the industry. The TiO_2_ and C1 fillers reduce cell size in the polyurethane matrix, positively reducing thermal conductivity and improving flame retardance while providing excellent quasi-static compressive properties and shape retention. The manufacture and analysis of polyurethane foams revealed six formulations of exceptional mechanical and chemical flexibility. Polyurethane foam is treated by the free expansion method. The most important new aspect in producing new polyurethane foam is the possibility of producing defect-free components or other nonreactive components. The basics of polyurethane foam manufacturing are reviewed, and new problems that the polyurethane industry is expected to face soon are addressed to improve the quality. Experiments have shown that polyurethane foams have superior shock absorption and heat resistance when the filler distribution is homogeneous in the polymer matrix. In microstructure studies, polyurethane foams can be represented as dispersed fillings in a bubble distribution integrated into the stored polyurethane foam material. The results indicate that foam strengthening in the nonlinear field is ineffective when injected fillers are larger than particle size. In addition, the successful replacement of toxic substances in the production of polyurethane foam can reduce its cost and impact on the environment. The production of polyurethane foam may also mitigate potential traffic accidents and improve the sustainability of the environmentally friendly polymer industry.

### 1.2. Manuscript content

- The viscoelastic properties of polyurethane foam are highly dependent on the density, cell structure, ratios of closed and open cells, and the concentration of the fillers.
- All polyurethane foams deteriorate during the thermal disintegration of the hard segments, while the soft segments degrade during the temperature decomposition.
- The used mineral fillers have an average decomposition endothermically, and layers often create a physical barrier cover due to their shell-like shape, generating the so-called “labyrinth effect”.
- The quasi-static compression test of the polyurethane foam produces shock-bearing energy.
- The DMA device helped detect the developed polyurethane foams dynamic properties. As a result, the polyurethane foam has had enough time to relax via the β-strength increase and broad peak of Tg.
- In microstructure studies, polyurethane foams can be represented as dispersed fillers in a bubble distribution embedded in the buffered polyurethane foam material.
- The results indicate that foam strengthening in the nonlinear field is ineffective when the injected filler is larger than the particle size.

## 2. Experiments

### 2.1. Materials

The foam comprises a polyurethane matrix that contains polyols, a surfactant, catalysts, and mineral fillings. This matrix corresponds to a polyol P0010 provided by Confortchem and is a polyether polyol (POPE) grafted by 10% styrene-acrylonitrile (SAN) chains. At RT, its density is 1.1 g cm^−3^, and its viscosity is 5.842 Pas. Confortchem supplies the silicone surfactant L-580. The crosslinking agent of the chain extensor is glycerol (GCO). It helps synthesize a crosslinked PUR, which implies bypasses between the macromolecular chains taken from PanReac. The polymeric diphenylmethane diisocyanate (PMDI) was provided by BASF. The catalyst used by Niax is triethylenediamine (A-33), a potent amine catalytic agent. Its function is to accelerate the chain polymerization reaction and regulate the kinetics of response during the expansion of the foam. The blowing agents are dichloromethane, which AcroSeal bestowed. The selected colouring agent (AO) is a commodity used in and utilized in the polyurethane industry. It was acquired from the Big TEGO paste manufacturer, Evonik Industries. The plasticizer used is polyethylene glycol (PEG), which Interchimie acquired.

Bental supplies the filler with calcium carbonate C1, which has a high degree of polydispersity in its size range. The distribution spans distances ranging from 1 to 190 lm. Titanium dioxide (TiO_2_) was obtained from VWR PROLABO. The titanium dioxide size distribution ranges from 0.7 to 19 lm and has an apex of 3 lm. As seen, Table 1 contains these parameters, in addition to densities, articular surfaces, the average diameter, and the characteristics of the mineral fillers used (see Table 1).

Foam polyurethane is produced by reacting with a polyol matrix with a methylene diphenyl diisocyanate (PMDI). It forms urethane linkages when it reacts with polyols [26]. At the point of foam expansion, the water supplied when the polyol reacts with the MDI and carbon dioxide is formed.

### 2.2. Elaboration of polyurethane foams (PUR)

The constituents of the polyol matrix A (Polyol, glycerol, silicone L-580, catalyst A-33, dichloromethane, polyethylene glycol) are combined at 700 rpm within 30 s using a mixer by Rayneri with a rotatable blade. This mixture is then desiccated for 24 h, draining the leftover water through a tiny filter. The metal charges are maintained in a dry oven for 24 h to flush out the absorbed water on the surface. Regarding calcium carbonate, the drying temperature is 100 °C to avoid all changes in its chemistry, and the temperature for titanium dioxide and montmorillonite is 50 and 60 °C, respectively. The volume/ cc fraction of the crystallized filler was incorporated into the polyol array depending on the type of polyurethane foam developed.

Component A is mixed with mineral fillers, which are combined at 1600 rpm for 15 min. These mixtures are prepared in the presence of N2 gas to prevent water from entering through the air’s humidity. The specimen is then emptied into empty Teflon cups, and the mixture is left to rest for 24 h to get rid of air bubbles that entered the resin throughout mixing.

We combine component A with component B, adding polyisocyanate to the matrix of polyols. The mixture (polyisocyanate-suspension) was stirred at 1600 rpm for 30 s, and then the mixture was put into a cylindrical mould. Foaming happens afterwards, allowing free expansion within this mould. The stretching phase continues for around 3 min, and then the anagen phase (polymerization and crosslinking) begins. The foam is then loosened after 30 min. The primary water concentrate is the main factor controlling the density of the final polyurethane foam, and its assessment and control are essential. The obtained mass is then chopped into small pieces with (68 × 68) mm cylindrical dimensions. Bulk density (d) is measured by measuring the volume and mass of these masses. Support information (SI) represents the appearance of the final foam after the preparation and levelling process (as seen in Figure S1).

We have provided a suitable environment for the preparation of samples. In addition, we have acquired new materials of international quality to reach the optimal formula for the developed polyurethane foam (as seen in Table 2, which summarises all the materials used in the preparation process).

### 2.3. Physical-chemical characterization

#### 2.3.1. Differential scanning calorimetry (DSC)

Differential calorimetry scanning studies were done utilizing nitrogen flow. DSC uses nitrogen dehydration to keep polymers from degrading at high temperatures. The temperature of indium and the enthalpy of fusion have been computed. The nonhermetic aluminium capsules were utilized for sample preparation and comparison. The capsule is completely covered with 3–5 mg of the sample to achieve the desired heating. The capsule has been pressed. The capsules are evaluated for DSC. The next series to choose is the 20 °C equilibrium heating, which heats the initial ramp at a 10 °C/min rate up to 125 °C. The ramp was under −100 °C at a rated temperature of 10 °C/min. Second ramp heating at a temp rate of 10 °C/min besides approximately 250 °C, 200 °C, or 150 °C, based on the TGA denaturation heat. In the second ramp, the temperature increases by 10 °C/min to roughly 250 °C or 200 °C depending on TGA denaturation.

#### 2.3.2. Thermogravimetric analysis (TGA)

The TGA was done using a TA Instruments Q50 under an N_2_ flow. The sample weighs between 3 and 4 mg. Thermal degradation occurs in the presence of inert or oxidizing gases. The pieces are heated at a rated temperature of 10 °C/min between 20 and 600 °C.

### 2.4. Mechanical tests

#### 2.4.1. Dynamic mechanical analysis (DMA)

We tested six proven samples, formulations prepared for shock absorption and firefighting, and compared them with each other. In this case, the tested specimens will be rectangular specimens with 5 mm edges. The dynamic mechanical analyzers–TA Instruments and polyurethane foam samples are ready for testing (see Figure S2) in SI. The dimensions of the test specimen were chosen as such, as these are the maximum conceivable dimensions to be able to apply a sufficient level of creep force (0.25 MPa, GTT specification). It is a reversed pendulum in torsion functioning in a forced harmonic domain at low frequencies between 10^−3^ and 3 Hz and temperatures ranging from −80 to 200 °C. According to preliminary studies, the humidity did not affect the mechanical qualities of the foam examined. The parameters used are as follows:

§ Temperature balayage method.
§ The applied force is 0.01 N, with an accuracy of 0.001 N, the frequency of 0.01 Hz, and the imposed deformations can be between 1 μm and 240 μm, with an accuracy of 0.1 μm and strain of 0.01%.
§ Temperature ranges from 80 to 200 °C in 5 °C increments. The frequency is 1 Hz with 20 steps. This sweep is performed at each temperature level.
§ Imposed displacement at 20 μm.
§ As previously stated, the displacement sensors are zeroed at the test’s beginning temperature, namely −80 °C.
§ Before doing any mechanical testing, the specimens are stored for 10 min at 70 °C (Renewal above Tg) and then for 24 h at 30 °C.

#### 2.4.2. Compression tests

Uniaxial compression testing was conducted using machine hydraulic press systems from 100 to 100,000 automated production lines. P. E. I. Ball bearing-guided is used to maintain parallel compression plateaus. This chamber is temperature-controlled, enabling it to conduct experiments between 80 and 200 °C to minimize barrel distortion and buckling, and 40 mm cylinders were used as test cylinders to ensure parallel contact surfaces were covered. They are lubricated with wax (molybdenum grease). At a temperature of 30 °C, the tests were done at a constant crossheading speed of 100 s^−1^. This test involves compressing the sample to 80% of its specific thickness. Nominal values are used to indicate strain and stress.

### 2.5. Microscopic characterization

#### 2.5.1. Scanning electron microscope (SEM)

We used an SEM JEOL-JSM 840SM-840 and a filament tensioner of 10 kV to characterize the cell microstructural and mesh size of the same polyurethane foam. The specimens are first shattered in fluid nitrogen. Following that, gold is applied to the surfaces to conduct them. The micrographs are images examined featuring pictures of software products to determine the particle size of the vacancies in the foam. After assuming sphericity in its shape, the pore sizes apparent are obtained. The Saltykov approach [27] permits the division of the actual width of the vacancies to be determined.

## 3. Results

### 3.1. Physical-chemical characterization

#### 3.1.1. Differential scanning calorimetry (DSC)

**Figure 1:**
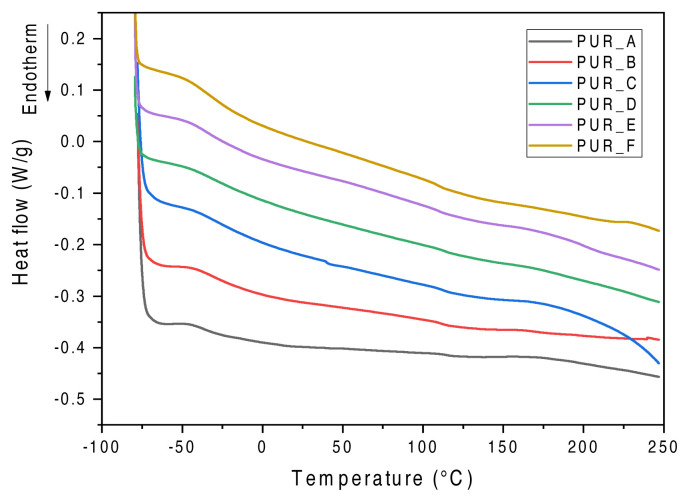
Tests by DSC for foams fostered by TiO_2_ and C1. T = −50 °C at 180 °C, ΔT = 10 °C min^−1^, N_2_.

DSC analysed polyurethane foams. We measured the glass transition temperature (Tg) using TA Universal Analysis software, which gives accurate results. We recorded the data (see Table 3). This indicates that foams containing vol.% Titanium dioxide (TiO_2_) and calcium carbonate C1 have increased thermal stability (see Figure 1). The higher Tg remains at 57 °C.

#### 3.1.2. Thermogravimetric analysis (TGA)

**Figure 2:**
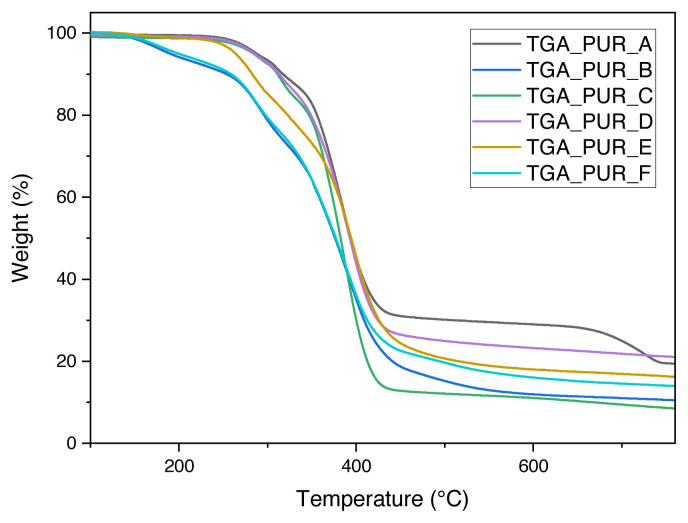
Progression of the % loss of mass about temperature for different polyurethane foams.

**Figure 3:**
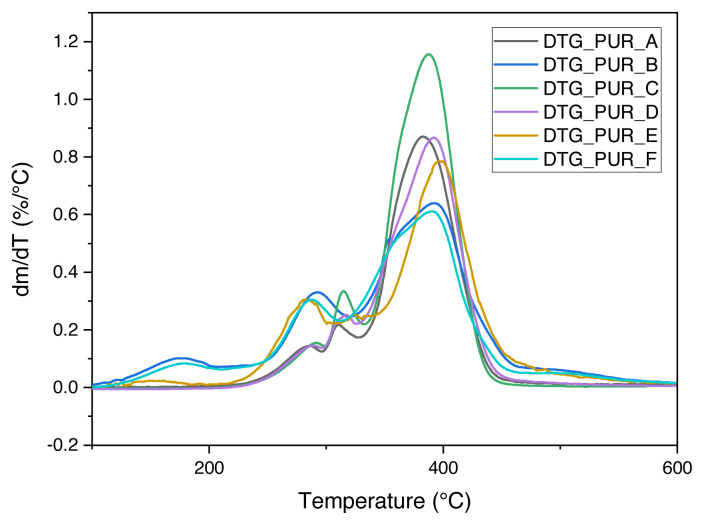
Differential curves (DTG) of polyurethane foam.

We present the results of TGA tests for six samples of polyurethane foam intended for shock absorption and firefighting, presenting the TGA percentage progression loss of mass at a temperature for different polyurethane foams (see Figure 2). At the same time, we show differential curves (DTG) for polyurethane foam (see Figure 3).

The summing-up of the experimental results of the thermal tests is indicated (Table 3).

Using the TGA curve as a base has demonstrated the percentage of the residual mass of polyurethane with different proportions of titanium dioxide and calcium carbonate. The temperature at which polyurethane foam breaks down has been determined to be between 190 and 600 °C. Calcium carbonate is decomposed at a temperature of between 680 and 875 °C, where carbon dioxide is emitted, and calcium oxide is produced. Whereas the temperature of decomposition of titanium dioxide is more than 700 °C, the thermal stability of polyurethane increases when a filler is added, and the specific gravity and toughness of polyurethane are also increased by adding the stuffing. The residual mass of polyurethane foam containing calcium carbonate is greater.

All polyurethane foams degrade through two distinct processes. The first thermal degradation step results from the cleavage of the urethane bonds. It results in the first degradation process occurring during the thermal disintegration of the hard segments, and the secondary degradation step occurs during the temperature decomposition of the soft segments.

Both the beginning temperature of decomposition (Td5%) and the maximum temperature of weight loss (Tdmax) of PUR A were lower than those of PUR F, which can be attributed to the nanoparticle-PMDI bonds being broken. The urethane linkages generated between polyether polyol P0010 and PMDI are less thermally stable. It has been proved that calcium carbonate and titanium dioxide were homogeneously distributed in all polyurethane samples as the percentage of the remaining mass corresponded to the group of the amount added.

The information collected during the research showed that the enhanced polymerization parameters could provide lot better results for the thermal characteristics of polyurethane. In our study, the maximum weight loss occurred at 28.82% at 383 °C. Compared to what Südemen and Önen [19] achieved, the weight loss was 50% between 392 and 401 °C for polyurethane and between 375 and 392 °C for poly(urethane-acrylate)s. The productivity of coal from poly(urethane-acrylates) increased after increasing the phosphorous content.

The theoretical and experimental values differ according to the polyurethane foam composition. For example, Kaya [28] concluded that BA acts as a barrier to preventing mass loss through thermal deterioration. The mass loss of virgin PUs decreased by 12%, 15%, and 21%. The coal layer, formed from rapid combustion, works as a preventative measure against the discharge of oxidizing gases and heat transmission to the inner layers. The advanced deterioration that occurs is beneficial for an excellent antiflammability effect. In contrast, BA adheres to the surface in the form of a thin coating that prevents the release of delayed combustion and toxic gases by reducing contact with oxygen and increasing thermal resistance.

Song et al. [29] designed and examined the heat stability of GOD-supported composite PVA films via nanotechnology and evaluated the heat stability parameters. Ti temperature at which 5 wt% of weight loss results, and Td is the temperature at which most weight loss occurs, steadily developing GOD contents. They concluded that GOD-2.0 and GOD-5.0 increased PVA of 264 °C to 272 °C and 290 °C, and Td of 286 °C to 355°C and 357 °C, respectively. They mean that 2.0 wt% of GOD leads an 8 °C rise in Ti and 69 °C in Td. In addition, this work shares our study a facile, bioinspired methodology for designing robust, rigid and thermoplastic polymeric materials used in electrical devices also tissue engineering, such as acritical skin for robots.

#### 3.1.3. Flame retardancy mechanism

**Figure 4:**
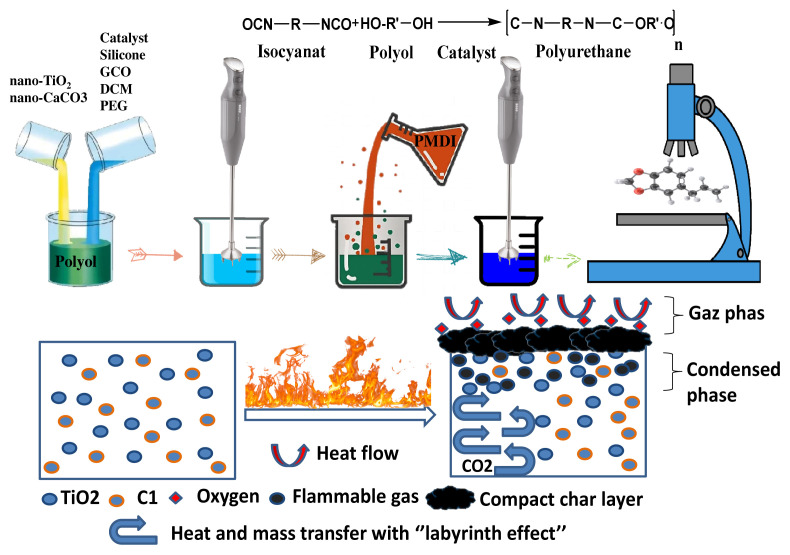
A possible antiflammability effect of action mechanism of titanium dioxide (TiO_2_) and calcium carbonate (C1) in polyurethane.

Delaying the pyrolysis method and reducing the temperature increase on the surface of the substance is achieved by adding additives with high thermic conductivity, which distribute the heat flowing of the flame throughout during the volume of the matter [30]. Moreover, the used fillers have an average decomposition endothermically, removing heat from the combustion operation prolonging the pyrolysis onset time. Mineral filler layers often create a physical barrier cover due to their shell-like shape, generating the so-called “labyrinth effect”. Titanium dioxide (TiO_2_) and calcium carbonate (C1) nanoparticles affect [31,32] the release of flame-retardant gases, for example, CO_2_, NH_3_, and H_2_O, resulting from their thermal decomposition, which simultaneously dilutes the flammable gases. Smouldering mixtures, resulting from cutting volatile pyrolysis constructed with noncombustible gases, which have a minor concentration of oxygen, are not capable of spontaneous combustion or continuous combustion [33]. Figure 4 illustrates the effects of titanium dioxide (TiO_2_) and calcium carbonate (C1) on the flammability of polyurethane.

### 3.2. Mechanical tests

#### 3.2.1. Dynamic mechanical analysis (DMA)

The elastic modulus G’ and viscous modulus G”, also known as tan modulus, were measured by DMA. Parallelepiped forms are used to test the samples (5 mm in width, 21 mm in length, and 1.6 mm in thickness). They endure a sinusoidal torsion, resulting in a dominating deformation (about 10^−3^, that is to say, in the linear field) at a frequency of HF f = 1 Hz. A ramped temperature was carried out throughout the testing from −80 to 200 °C with a heating rate of dT/dt of 3 °C/min [34]. Figure S3 in SI displays polyurethane foam’s mechanical dynamic analysis (DMA) results.

**Figure 5:**
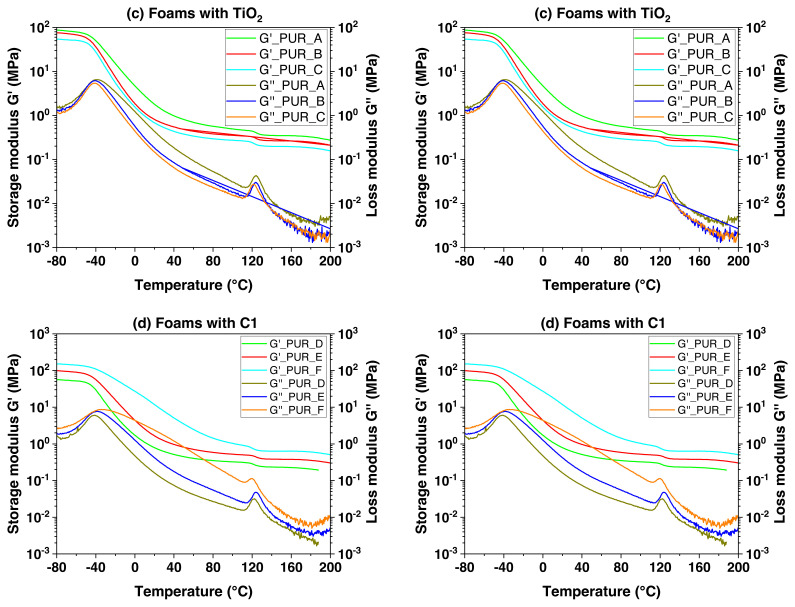
Tests by DMA for polyurethane foam rein forced by TiO_2_ and C1. T = −80 at 200 °C, f = 1 Hz, ΔT = 3 °C min^−1^.

Dynamic mechanical analysis measurements (see Figure 5) indicate that the temperature is similar to the significant relaxation, equivalent to 82 °C regardless of the intensity. At approximately 10 °C, a relatively large complement slack was discovered in low-density unfilled foam (d = 0.07). The second relaxation comes from developing domains from the reaction byproducts. For example, urea is produced when water reacts with isocyanates. Despite the presence of this complementing relaxation, excellent modelling of the unpacked foam’s viscoelastic behaviour under no matrix alteration indicates that this modest alteration is inconsequential. Other filled foams do not exhibit this subsequent relaxation. Gaskets are inserted to absorb the heat generated during the polymerization operation. A fraction of these byproducts may be reduced in kinetic production at temperatures over 100 °C, indicating that the treated foams have a comparable polymer matrix.

**Figure 6:**
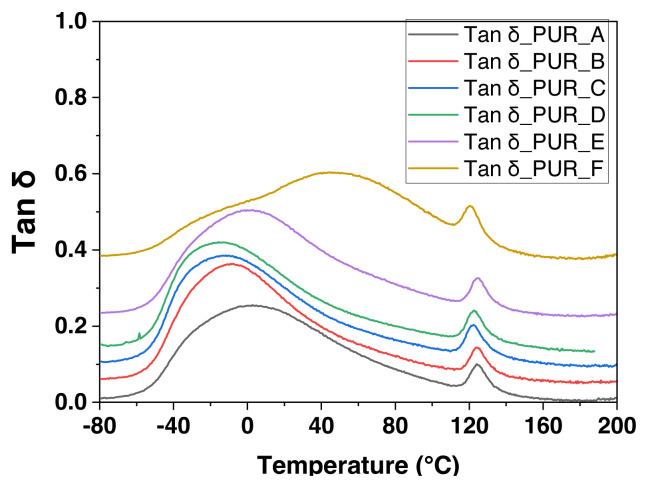
Tan δ curves: DMA test results.

The progress of the dynamic properties of polyurethane foam loaded with nanofills was monitored. Dynamic mechanical analysis (DMA) determined the G’ modulus development characteristic at the function temperature (see Figure 6). Curves of different densities appear parallel and are changed by an ingredient on the logarithmic scale. Whatever the filler, a decrease in the G’ modulus can be observed as the density decreases. TiO_2_ filled foam with proportional densities of 0.22 and 0.25. We noticed that the current values of G are lower than those of TiO_2_-filled foam. For extreme density, G’ values tend towards those of C1-filled foam. These test results show the impact of filler volume on the flexible conductivity of polyurethane foams: as the mean filler volume decreases, the G’ modulus values rise.

There may well be substantial mixing of the various particle molecules based upon their chemical structure, length, percentage of components, mode of production, and different processing stages, according to Cooper and Huh, 1971 [35]. Mechanical characteristics of polyurethane block polymers in dynamic conditions may cause the PUR to display specific glass transitions in the realms of hard and soft cutoff. Furthermore, crystallization can occur during both phases. Soft rubber bands impart elastomeric characteristics. The stiffer solid part domains, which remain united with a secondary hydroxyl group and possibly crystallization, serve as a crosslinked polymer for the rubber and the soft part matrix. At −60 °C, the transition to a lower temperature occurs and is associated with a stiff to a viscous characteristic. At the same time, the thermal transition temperature at approximately 135 °C is thought to be related to the breakdown of hydrogen-related van der Waals interactions between the rigid and elastic parts of the PUR. The modulus is less influenced by temperature between such two transition zones and is often abbreviated as the “plateau” modulus [36,37]. The most important results from the dynamic mechanical analysis (DMA) are seen in Table 4.

The viscoelastic behaviour of various polyurethane foam samples was investigated (Figures 5 and 6). The inflexion of G” at −34 °C for formulation 01 and −40 °C for formulation 02 corresponds to the peak G” of the loss modulus curves at comparable temperatures and with a tan δ as the peak loss factor. They are attributed to the relaxation of α or Tg, tracked by a region of the rubber plateau. The secondary peak in G” at 121 °C relates to the β transition for formulation 01 and 122 °C for formulation 02, which occurs as a result of the first movement of the molecular side-chain segment and may also have a strong PUR effect at ambient temperature, expanding the side chain’s range of motion [38]. As a result, the strength of the transition has increased [39].

The small peak β indicates resistance to the weak effects of PUR. Additional data, including accurate impact test results for parameters on and practically the relaxation, should be utilized to demonstrate the transition effect on impact-resistant behaviour [36]. What is interesting in the studied samples is the presence of two peaks, the first large and the second minor, in the first curve of tan δ at 20 °C for the formulation 01, and −10 °C for the formulation 02, probably due to the evaporation of water from permeable PUR that may accumulate during the load stress on the sample. The secondary peak at 121 °C shows an artefact due to the selection shrinking at a significant temperature.

Thinners are effective plastics in removing the limitations of amorphous segments and thus increasing movability due to increased free size (a decrease in Tβ). They also allow more confirmation of reorientation (an increase in β intensity) [36]. We will test their theory by pointing out the more substantial “rest peak in G”. We recorded the transition of Tg to a lower temperature, compared to the same materials tested by DSC at about −40 °C in the curve G’.

The glass transition temperature Tg was calculated using the tan δ curve’s peak height. The inclusion of 7.5 wt% TiO_2_ did not affect the glass transition temperature. But when you add 9.78 wt% of TiO_2_ mixture, move to the high-temperature range. Authors allow the elastic polyurethane foam to become plasticized. The effect of TiO_2_ on polyurethane foams may also be seen by the storage modulus curves that are temperature-dependent. In the instance of polyurethane foam modification using calcium carbonate nanoparticles C1, it is possible to see a shift in the glass transition temperature Tg to a higher range of temperatures, increasing the modified material’s stiffness.

The broader tan δ curve and its elevation to higher levels as the temperature −80 at 80 °C may be credited additionally to the polyurethane’s permeability due to its semicompatible morphology. It has been observed in previous studies that a comparable impact, manifested by an initial increase in the quantity of nanoclay up to a specific level and a subsequent drop after reaching this level [40], and a rise in the elastic constant by the effect of filling dispersion [41].

#### 3.2.2. Compression tests

**Figure 7:**
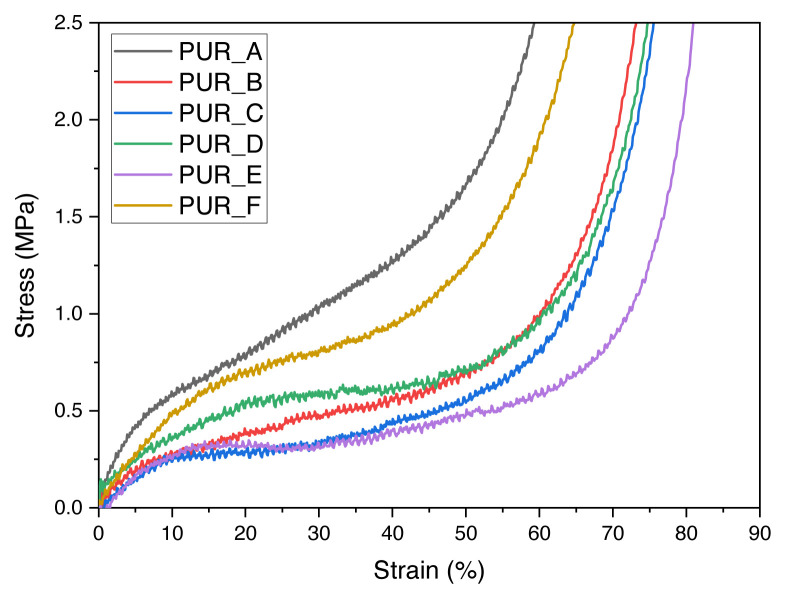
Compression curves of the prepared polyurethane foam.

Figure 7 illustrates the compressibility curves of polyurethane foams of various densities filled with titanium dioxide (TiO_2_) and calcium carbonate (C1). It is a characteristic of polyurethane foam, beginning with an elasticity domain and progressing via a condensation plateau, a hardness domain, and finally, a terminal rupture. The plateau is seen to occur at a consistent strain level of around 30%. As demonstrated by the instability of the curves, the C1-filled PUR_E foam appears to be further brittle (Figure 7). At any given stress level, all graphs demonstrate a reduction in strain value with decreasing density. The modulus of elasticity of compression for various samples and the slope of the compressive curve at its origin may be calculated.

As seen in Figure 7, a representative compression test demonstrates the evolution of the mechanical characteristics of TiO_2_ and C1 laden polyurethane foam. The foam samples exhibit relatively rapid production following the first linear load regime, and they show a long-sustained plateau area with a rise in stress at rising strain. The primary linear area determines the foam modulus. The large plateau region is attributed to the failure or twisting of the foam’s cell wall and is mentioned as “failure stress.” At lower densities, we see that the pressure following the plateau drops, resulting in a yield point-like behaviour. They show that the pressure increases as the polyurethane foam condenses [42]. We offer the results of mechanical tests (see Table 5).

### 3.3. Microscopic characterization

#### 3.3.1. Scanning electron microscope (SEM)

**Figure 8:**
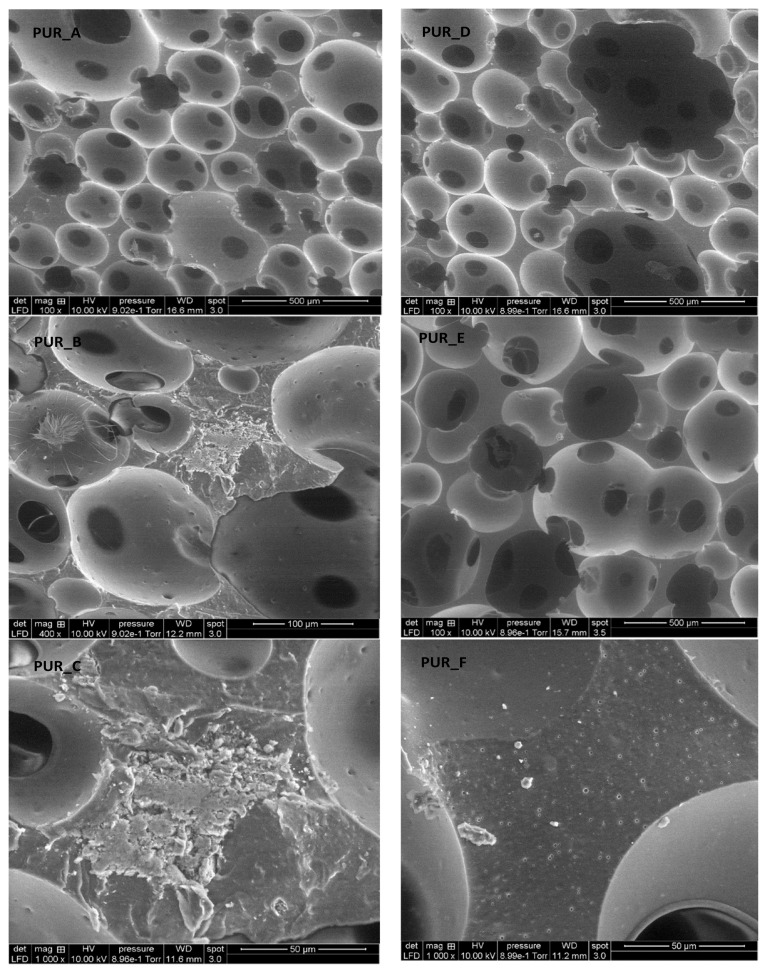
SEM micrographs show PUR pores’ distribution for formulation charge, TiO_2_, and C1.

**Figure 9:**
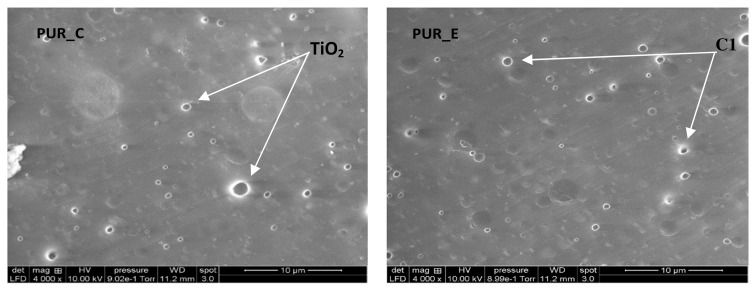
SEM micrographs showing the PUR pores and distribution of 10 μm of TiO_2_ and C1 particles in PUR foams.

SEM micrographs of foams reinforced with titanium oxide (TiO_2_) and calcium carbonate (C1) (see Figure 8). The cells appear mainly spherical and half-open in the studied density range (0.18 at 0.34). The particles are moderately distributed in the polyurethane matrix, regardless of the type of filler and foam density. The apparent distribution of cells-sized as a roll of polyurethane foam density was determined via analysis of images. Estimating using the Saltykov technique [27] permitted us to infer the proper size of the dispersion (Figure 9). The padding does not modify any cell structure but reduces its size dispersion. In the examined density series, the size of the cell L, signalled in micrometres, can be approximately tuned as determined via the linear function (1):


(1) L=A×B+C

Wherever C corresponds to the proportional density of the foams and A and B, they are adjustment factors pronounced in micrometres by the least square linear regression technique. A and B for PU filled with C1 and PU served with TiO_2_, respectively, by Burgaz [43]. Furthermore, Siegmann et al. [14] did not comply with it, investigated three classes of fillers: graphite particles, fibreglass, and glass beads. It is worth noting that the filler concentration, given as a mass fraction, is less than 30% in all of these jobs, and the volume fraction is lower than in our research. The combination, the period of the loaded suspension with isocyanates, mould size, and components all varied.

For us, the lower cell size emerges as likely due to the result of the filler nucleus. Moreover, that impact can be improved by water remaining on the surface of the particles, causing the release of additional carbon dioxide. However, contrary to our expectations, particle size distribution does not affect cell volume distribution. Instead, they indicate that the result of the nucleus, if present, is out of proportion to the particle’s surface.

The wrinkles on the cell walls indicate the contact points between neighbouring cells. These interaction regions are composed of thin, continuous polymeric membranes that function as cell walls. The union of many cells leads to the development a cellular scaffold, which serves as the foam’s primary structural component. An enlarged version of a typical polymer stent demonstrates this critical characteristic (see Figure 9). The high magnification picture indicates that the cellular walls are extremely thin, just a few micrometres thick, and therefore that the bulk of the polymerization is contained inside the supports. By increasing the density of the polyurethane foam, smaller cells are obtained. The structure retains its homogeneity and maintains a consistent cell diameter. The walls of the cells are typically thicker at greater densities.

**Figure 10:**
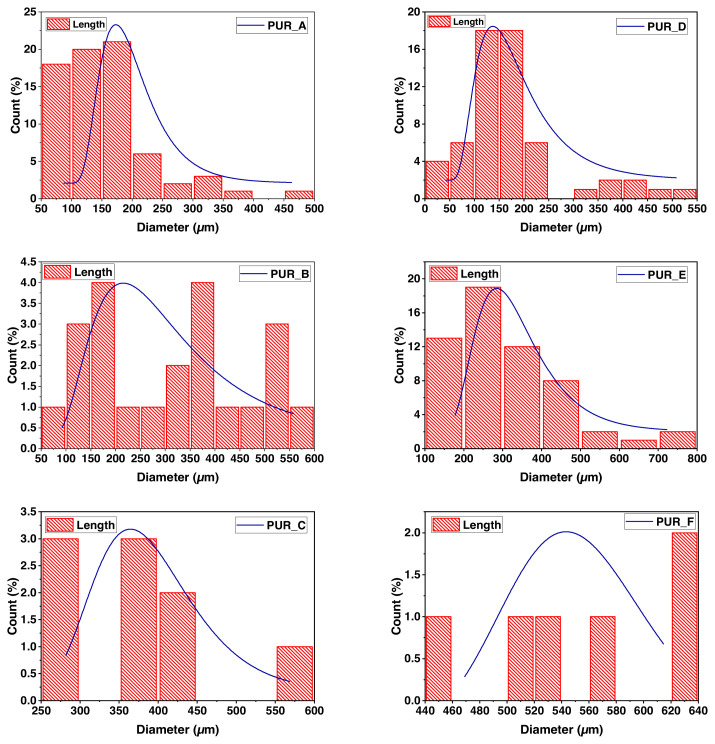
A histogram of the distribution of the mean pore diameter of the various produced PUR.

Because the polymer supports are limited by open foam cells, the TiO_2_ and C1 powder particles are computed separately (see Figure 10). As demonstrated, increasing the filler percentage resulted in a marginally greater density of particles inside the supports and cell walls. Additionally, a quantity of metal powder detached from the polymer during fracture, creating tiny dimples.

Figure 10 illustrates the normal distribution of foam cells. The faint structures on the cell walls are filler particles that lie just under the inner cell wall-free surfaces. They are all filler particles that lie just under the inner cell wall’s free surface. We analysed the particle size for the SEM micrograph (histogram) using ImageJ software, and we got the results (Figure 10).

The average pore diameter distribution for the various PUR was produced (see Figure 10). The vacuum content in the foam cells is extensive compared to the used filler. We observe the cells distributed in a bubble shape. The cell wall covers the nanofilling with calcium carbonate and titanium dioxide, where the area of coverage of these fillings is greater than the area of the foam’s pores. The result is that the fillers’ properties determine the foam properties. Its characteristics are crystal structure, particle morphology, and size.

There were no accumulations of calcium carbonate and titanium dioxide particles, i.e. their concentration increased to the ratio of nanoparticles (0.01 m). They have a large area about their size, spread across the windows of the gigantic cells of polyurethane foam. At these concentrations, the fillers form a large number of tiny cells capable of interacting with a large number of soft components on a large number of polymer cell windows. They significantly increase the hardness, as titanium dioxide contains 9.78 wt% and calcium carbonate contains 8.85 wt%.

The soft component reacts with the polyurethane matrix, reducing its mobility and the rate of change in the plateau edge region. That boosts the polyurethane foam’s toughness dramatically. Additionally, gas expansion improves thermal stability and flexible cellular nucleation by generating a protective barrier.

**Figure 11:**
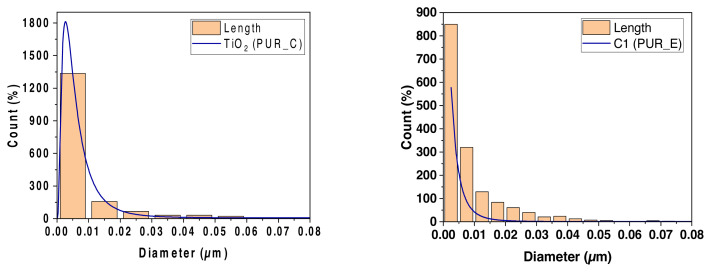
A histogram of the distribution of the mean diameter of nanoparticles of TiO_2_ and C1 in the foam matrix PUR_E and PUR_C.

Different nucleation processes produce a variety of initial gas cell sizes. The pressure inside these cells exceeds the saturation liquid with gas strength. As it turns out, when a distribution of cell sizes exists, the smaller bubbles are subjected to significantly greater pressures than the more giant cells, which face more significant forces from the large expanding bubble. The filler dispersion in a polyurethane foam backing (Figure 11).

As the filled polyurethane foam develops, cells form as gas bubbles develop. The liquid layer becomes thinner within each cell, causing cell window drainage. A critical threshold is achieved when different instabilities set in, leading these cell membranes to break. The breach causes cell coalescence, raising the average size of the cells and widening the cell size dispersion.

Because air bubbles are superior to the polymer solution medium in terms of the disseminated weight, they have a penchant for rising to the foam’s surface. This process results in the bubbles rising and cream fast at the top of an air foaming system with low viscosity and giant bubbles [44]. Another process closely linked to crime is flocculation, which occurs when particles cluster and stay together. Flocculation is a natural process that increases particle/bubble size, which increases creaming rates.

#### 3.3.2. Descriptive statistics

We summarize the distribution of the polyurethane foam cells and the filler (see Table 6). The SEM micrographs were employed to quantify TiO_2_ and C1 particle distribution in polyurethane foam. The polyurethane foam’s microstructured surface containing TiO_2_ and C1 particles reveals a multilayer structure that is a very compact network structure due to the mutual networking of the polyurethane foam particles. The polyurethane foam surface also had a specified microstructure and nanocomposite synthesis. As shown in Table 6, PUR/TiO_2_ and PUR/C1 nanocomposites have an average size of 9 nm, and these results confirm prior findings.

### 3.4. Mechanical behaviour modelling

**Figure 12:**
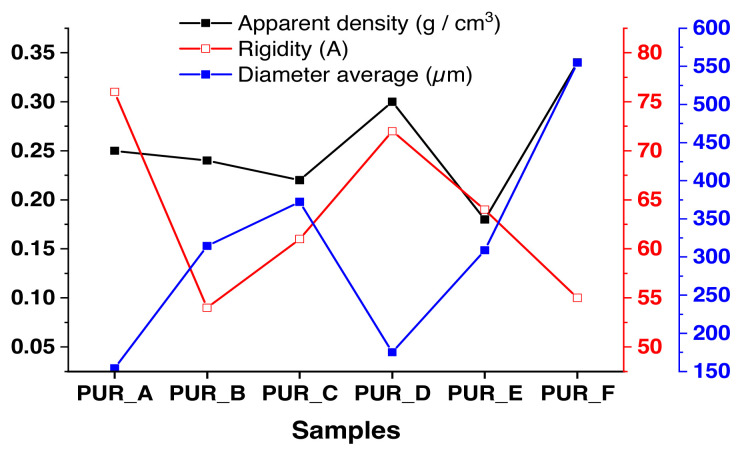
Curves with variable rigidity and bulk density with PUR are based on their additive material.

The infrastructure we have reached using the electron microscope SIM has physical-mechanical characteristics to obtain an ideal model. However, it deserves more digging, and from this point of view, it is necessary to search for a mathematical-physical model. That combines all computational dimensions and experimental results and compares them to scientists’ latest findings in the field of polymeric foams. For example, the corresponding (Figure 12) combines the polyurethane foam cell’s bulk density, hardness, and diameter. From this point, we will start this study.

**Figure 13:**
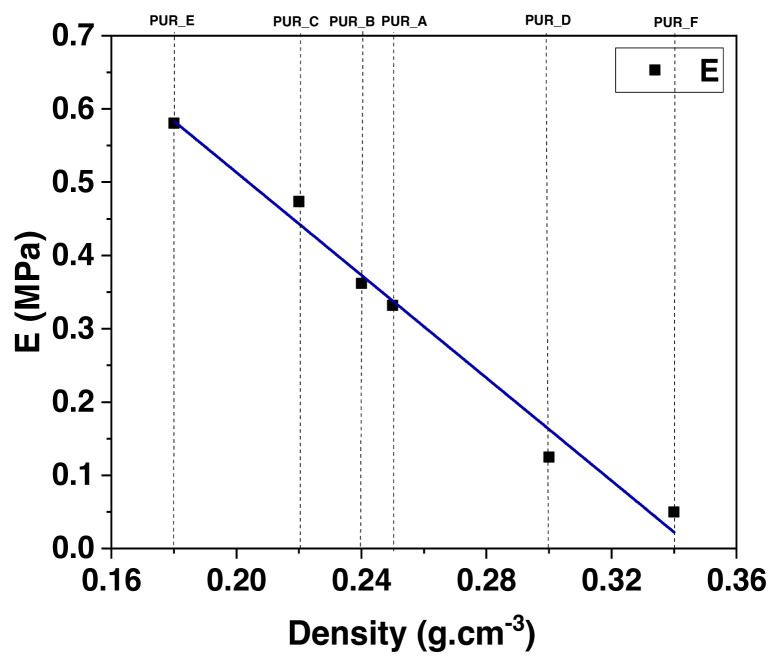
Modulus dependency on density in compression.

Modulus models are compared side by side, obtained from compression experiments and based on polyurethane foam density (see Figure 13). All the figures can fit into an expressiveness based on the power-law concerning the model’s density, in this way: E_∝_(ρ)n, E is the polyurethane foam’s Young’s modulus, ρ is the polyurethane foam’s density, as well as n, which symbolizes the density proponent. For example, the data in Figure 13 is well suited to a density proponent of n = 1.7 over the density range shown.

**Figure 14:**
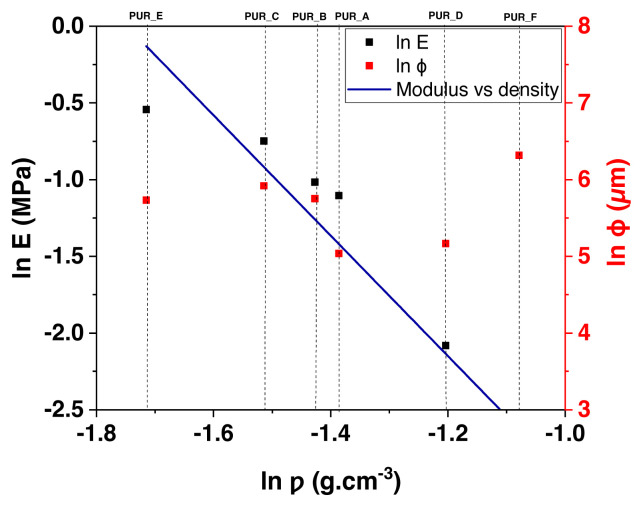
The log plot illustrates the power-law relationship between the modulus and the diameter of the cell polyurethane foam as the relative density.

Similar data as mentioned above on the logarithm of values with a density measured as that of polyurethane foam are compared to a solid polymer of 1.2 g/cm^3^ (see Figure 14). Therefore, a sample with an average density equals a completely dense large polymeric foam. Furthermore, the data is shown as a single direction with a ramp of 1.6, confirming the force-law relationship and the intersection of the most favourable data curve with the coordinates of ρ_PUR_ = 1, which may be called the polymer-solid modulus. Thus, we obtain a 2 GPa modulus, which falls within the stated range of PU values of 1.5 to 2.5 GPa [45].

**Figure 15:**
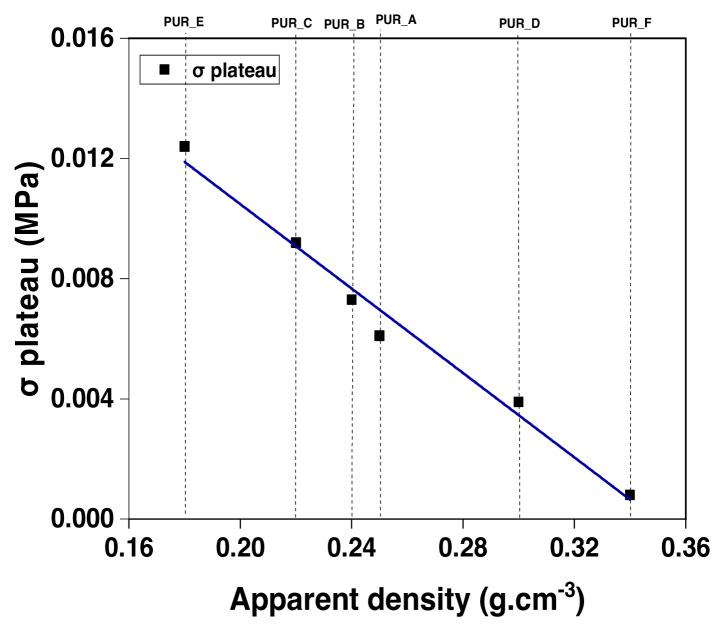
The evolution of the stress at the plateau’s beginning as a function of the total density as determined by compressive tests on polyurethane foam reinforced foams.

**Figure 16:**
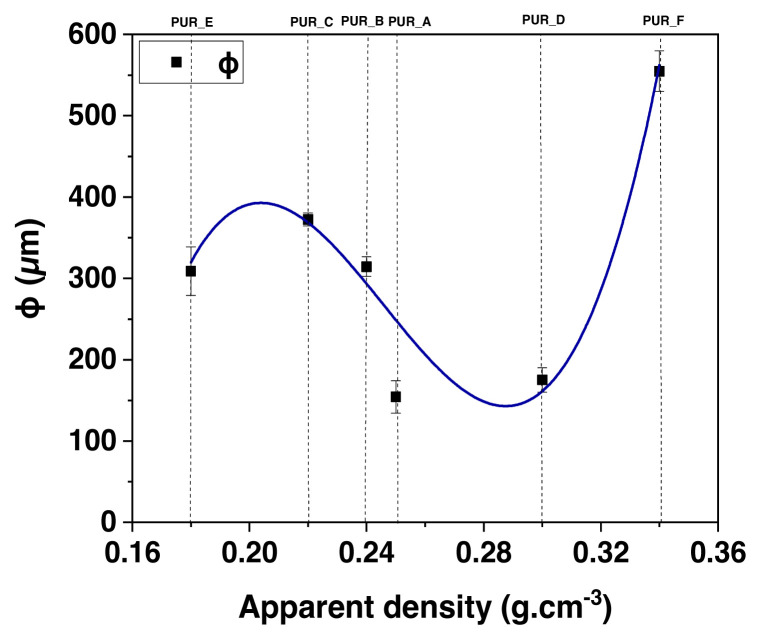
The mean cell diameter of the cell polyurethane foam is the relative density.

**Figure 17:**
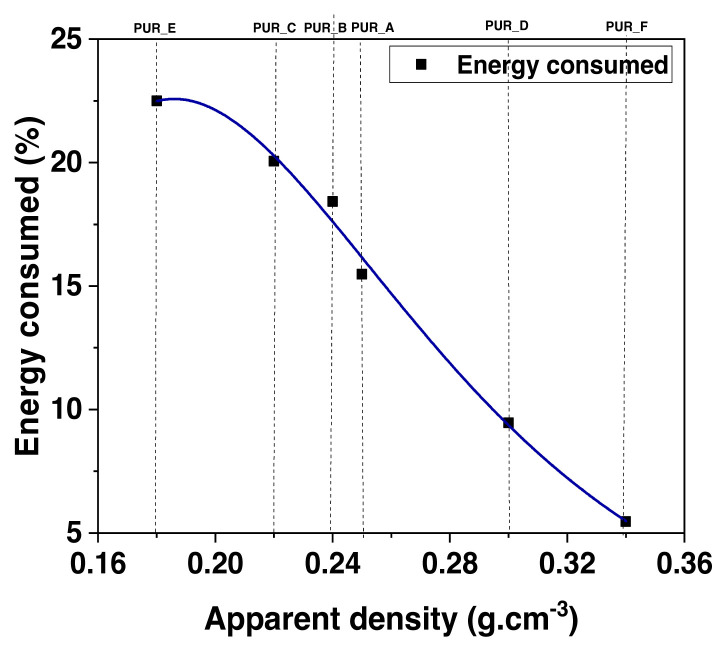
The energy consumed according to the apparent density of polyurethane foams.

The junction of the best-fit graph is used to determine the solid polymer modulus. The breakdown stress at the plateau of foam samples is proven as a proportion of density (Figures 15 and 16). They are crucial for the impact-mitigation design of polyurethane foams because they indicate the start of the structural instability of the polyurethane foam microstructure [46]. It also shows power-law dependency concerning the polyurethane foam’s density, notwithstanding that the exponent of density 2 is somewhat more than the value indicated by the modulus of elasticity (see Figure 17).

## 4. Conclusion

PUR reinforced with TiO2 and C1 was examined for shock absorption and thermal insulation. According to this study, the thermal insulation of foams can be affected by adding mineral fillers. In addition, TiO2 and C1 resulted in smaller cells, lowering heat conductivity. The DSC findings showed that foams with high TiO2 and C1 content enhanced thermal stability, and the higher Tg remains at 57 °C. Foaming polyurethane is a complicated process with numerous variables. TGA revealed that polyurethane foam decomposes between 190 and 600 °C. Adding a filler to polyurethane improves thermal stability. As a result, the leftover C1 polyurethane foam mass is bigger. The research revealed that altered polymerization settings might improve polyurethane’s thermal characteristics. For example, at 383 °C, the greatest weight loss was 28.82%. TiO2 and C1 also influence the thermal breakdown of flame retardants such as CO2, NH3, and H2O, which dilute flammable gasses. As a result, cutting volatile pyrolysis produces combustion mixes of noncombustible gasses with low oxygen concentrations that are not capable of spontaneous or continuous burning. In addition, the morphology and thermal insulation capabilities of the foams were not harmed by this change.

The dynamic mechanical analysis DMA device helped us Tβ was determined, and the initial derivation of G’ or dG/ dT was obtained. The broad peak of Tg in DMA indicates sufficient time for the polyurethane foam to relax through the β-strength increase. The balance between G’ and G” must be controlled to improve the characteristics required to create the necessary hardness and relieve stress at different temperatures. The broadening of the curve’s peak (tanδ) in the Tg range results from increased fillers in polyurethane foam. They can be linked to the glassy phase of the utilized stuffing, inhibiting polyurethane foam chain mobility. These test results show the impact of filler volume on the flexible conductivity of polyurethane foams: as the mean filler volume decreases, the G’ modulus values rise.

Compression tests on polyurethane foam are represented by curves that begin with an elasticity domain and progress via a condensation plateau, a hardness domain, and finally, a terminal rupture. The plateau is observed at a constant strain level of roughly 30%, and the C1-filled PUR E foam seems to be even more brittle. However, all graphs show a decrease in strain value as density decreases at any given stress level.

SEM micrographs reveal that the cells in the examined density range (0.18 at 0.34) are mostly spherical and half-opened. The particles are evenly dispersed throughout the polyurethane matrix regardless of filler and foam density. We used image analysis to evaluate the apparent distribution of cell sizes and the filling in a roll of polyurethane foam. As a result, the quality of the fillers dictates the characteristics of the foam.

A physical model of polymeric foams is based on the model’s density power-law. The data is presented in one direction with a slope of 1.6, which confirms the force-law relationship and the intersection of the most favourable data curve with coordinates ρPUR = 1, which may be called the solid polymer modulus. The breakdown stress at the plateau of the foam samples comes as a ratio of density. They are essential for the mitigation design of polyurethane foam because they indicate the onset of structural instability in the polyurethane foam microstructure.

## Support information

Figure S1 Presentation of the final foam after the preparation and levelling process.

Figure S2 a) Dynamic mechanical analyzers–TA Instruments. b) Polyurethane foams samples are ready for testing.

Figure S3 Mechanical dynamic analysis (DMA) results of polyurethane foam.

## Figures and Tables

**Table 1:** Physical and chemical properties of used mineral fillings.

Mineral fillers	Average diameter (μm)	Density (g/cm^3^)	Specific surfaces (m^2^/g)	Poisson coefficient	Modulus (GPa)
Titanium dioxide	0.01	4.23	8.23	0.29	151
Calcium carbonate	0.01	2.7	0.4	0.27	14

**Table 2:** Antishock and heat-resistant polyurethane foam components.

Formulations	Foams	A						B		C
		Polyol POPE (wt, %)	Glycerol GCO (wt, %)	Silicone L-580 (wt, %)	Catalyst A-33 (wt, %)	Dichloromethane DCM (wt, %)	Dibutyl phthalates DBP (wt, %)	Titanium dioxide TiO_2_ (wt, %)	Calcium carbonate C1 (wt, %)	Polymeric diphenylmethane diisocyanate PMDI (wt, %)
**01**	**PUR_A**	60	1.38	0.83	0.93	0.73	1.35	9.78	0	25
	**PUR_B**	60	2.19	2.07	1.26	0.45	1.51	7.52	0	25
	**PUR_C**	60	2.15	1.46	1.26	0.6	2	7.53	0	25
**0**2	**PUR_D**	60	2.29	1.54	1	0.4	0.92	0	8.85	25
	**PUR_E**	60	2.28	1.24	1.79	1.22	0.69	0	7.78	25
	**PUR_F**	60	1.01	2.75	1.76	0.65	1.31	0	7.52	25

**Table 3:** Melting temperatures and glass transition of our polyurethane foams as determined by DSC (10 K min^−1^).

Foams	Tg (°C)	Td_max_ (°C)	Residue %	Td_5%_ (°C)
**PUR_A**	3	383	28.82	287
**PUR_B**	−23	392	10.36	183
**PUR_C**	−24	386	9.74	287
**PUR_D**	−28	392	21.17	282
**PUR_E**	−30	399	16.21	263
**PUR_F**	−32	391	14.24	191

**Table 4:** Test results DMA.

Foams	Tα °C	T_β_ °C	Tan δ max
**PUR_A**	−39.77	124.18	124.69
**PUR_B**	−41.36	123.93	123.93
**PUR_C**	−42.61	121.08	122.24
**PUR_D**	−42.85	121.72	122.25
**PUR_E**	−38.60	123.68	124.26
**PUR_F**	−36.48	119.77	120.35

**Table 5:** Characteristics of samples before and after mechanical tests.

Foams	E (MPa)	Plateau stress (MPa)	Deformation max (ɛ_max_)	Shore A (A)	Average consumed energy by Charpy (J)	Density (g/cm^3^) at 0 %	Density after compression (g/cm^3^) at 0 %	Length compression (mm)	Diameter compression (mm)	Weight of PU foams (mg)	Weight loss after compression (mg)	Mass loss after compression (% mg/mg)
**PUR_A**	0.33	0.0061	85.15	76	15.48	0.25	0.25	33.6	33.55	7.56	0.06	0.83
**PUR_B**	0.36	0.0073	85.11	54	18.43	0.24	0.24	33.56	33.5	7.09	0.03	0.36
**PUR_C**	0.47	0.0092	87.41	61	20.06	0.22	0.22	33.15	33.1	6.32	0.09	1.51
**PUR_D**	0.12	0.0039	87.6	72	9.46	0.30	0.23	32.3	32	5.95	0.06	0.95
**PUR_E**	0.58	0.0124	98.18	64	22.5	0.18	0.17	32.66	32.1	4.65	0.004	0.1
**PUR_F**	0.05	8E-4	85.81	55	5.46	0.34	0.33	32.4	32.48	9.22	0.1	1.13

**Table 6:** Calculation of radii values of polyurethane foam cells and filler material.

Foams	Dimensions	Minimum	Maximum	Mean (average)
**PUR_**A	**Diameter (μm)**	53.74	451.05	154.25
	**Area (μm** **^2^** **)**	3015	104184	23428.96
**PUR_B**	**Diameter (μm)**	90.44	586.01	314.45
	**Area (μm** **^2^** **)**	8776	197072	70367.9
**PUR_C**	**Diameter (μm)**	251.09	597.15	372.28
	**Area (μm** **^2^** **)**	24861	97452	63900.5
**PUR_D**	**Diameter (μm)**	42.52	501.95	175.17
	**Area (μm** **^2^** **)**	6204	146496	27397.07
**PUR_E**	**Diameter (μm)**	132.39	782.56	308.88
	**Area (μm** **^2^** **)**	16115.52	299073.25	72452.40
**PUR_F**	**Diameter (μm)**	454.23	634.19	554.75
	**Area (μm** **^2^** **)**	33541	158687	106450.75
**TiO** **_2_**	**Diameter (μm)**	0.001	0.18	0.009
	**Area (μm** **^2^** **)**	0.001	26.47	0.23
**C1**	**Diameter (μm)**	0.001	0.08	0.009
	**Area (μm** **^2^** **)**	0.001	5.55	0.15
