# Supplementary figures and images for: A new study of dynamic mechanical analysis and the microstructure of polyurethane foams filled
Source: Turk J Chem. 2022 Feb 23;46(3):814–34. doi: 10.55730/1300-0527.3371 (PMC10503975; doi:10.55730/1300-0527.3371)

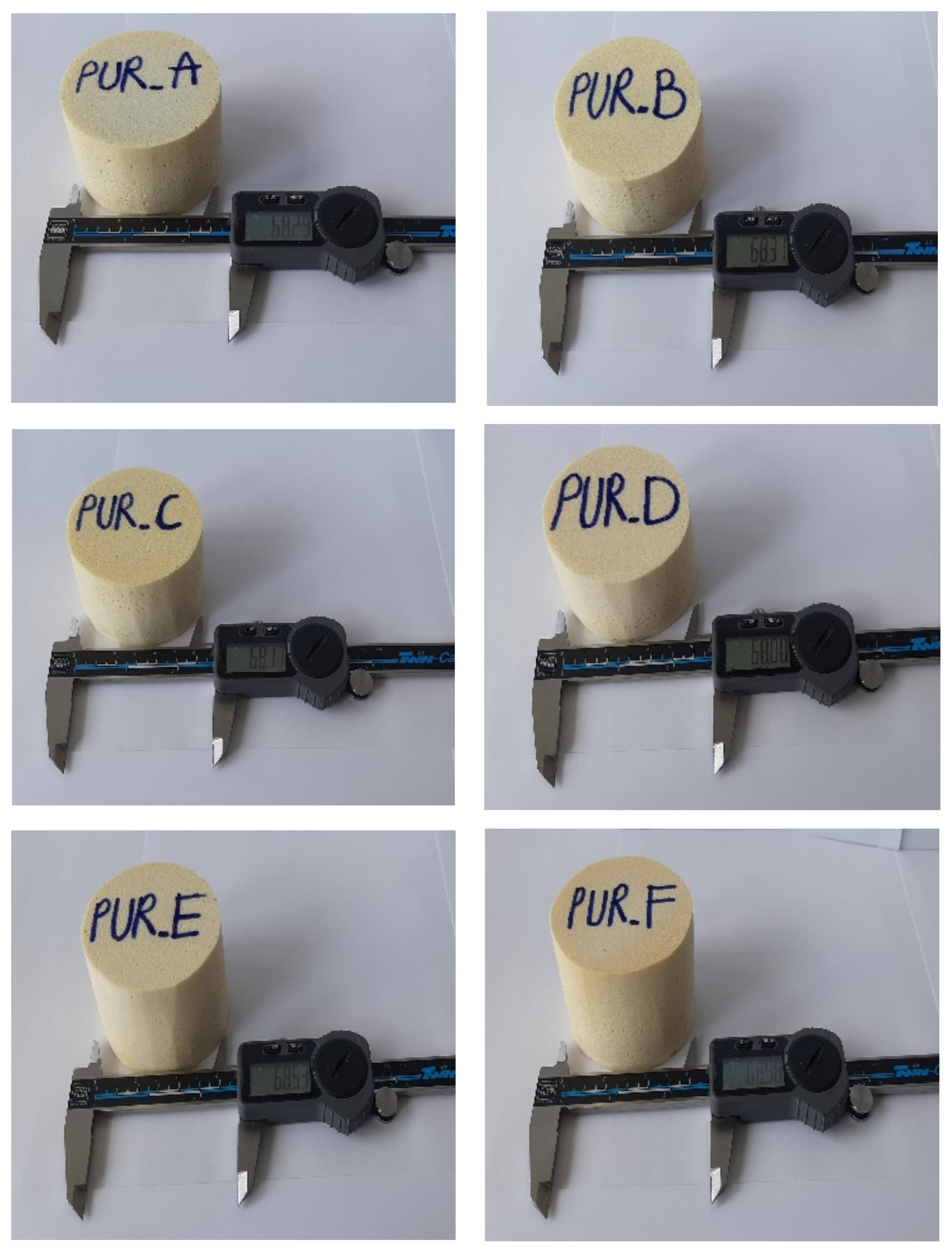

Supplement: Figure S1 — Presentation of the final foam after the preparation and levelling process. [file turkjchem-46-3-814s1.tif]

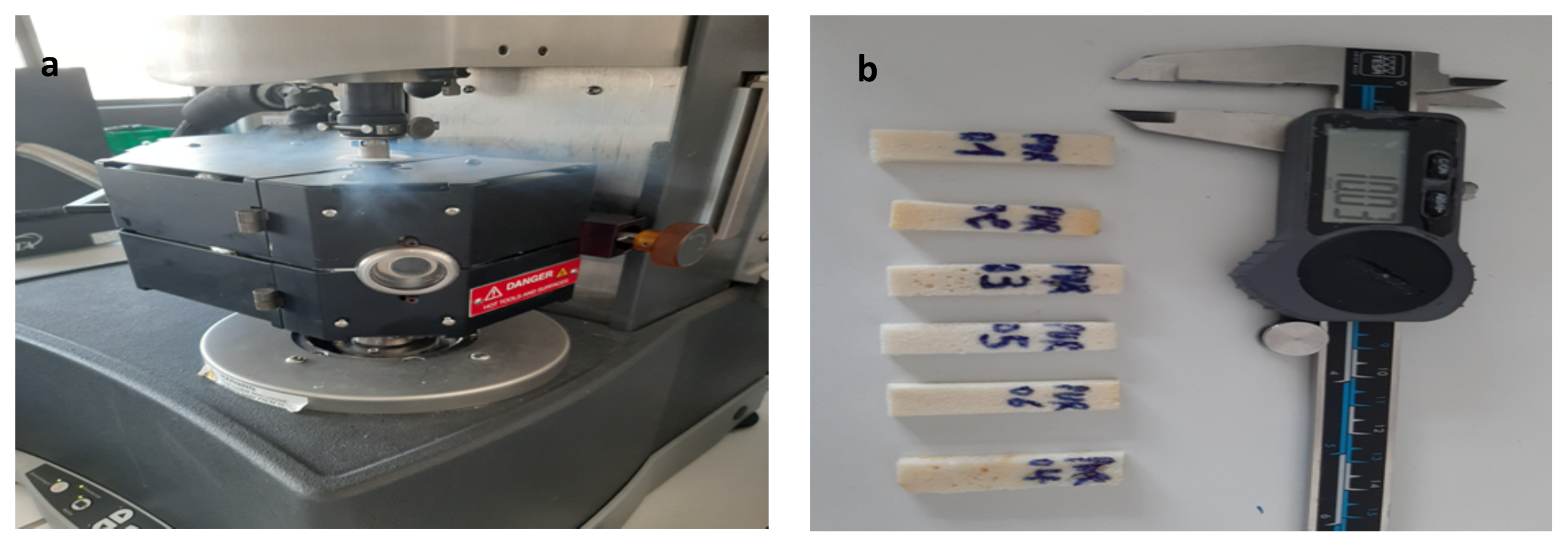

Supplement: Figure S2 — a) Dynamic mechanical analyzers–TA Instruments. b) Polyurethane foams samples are ready for testing. [file turkjchem-46-3-814s2.tif]

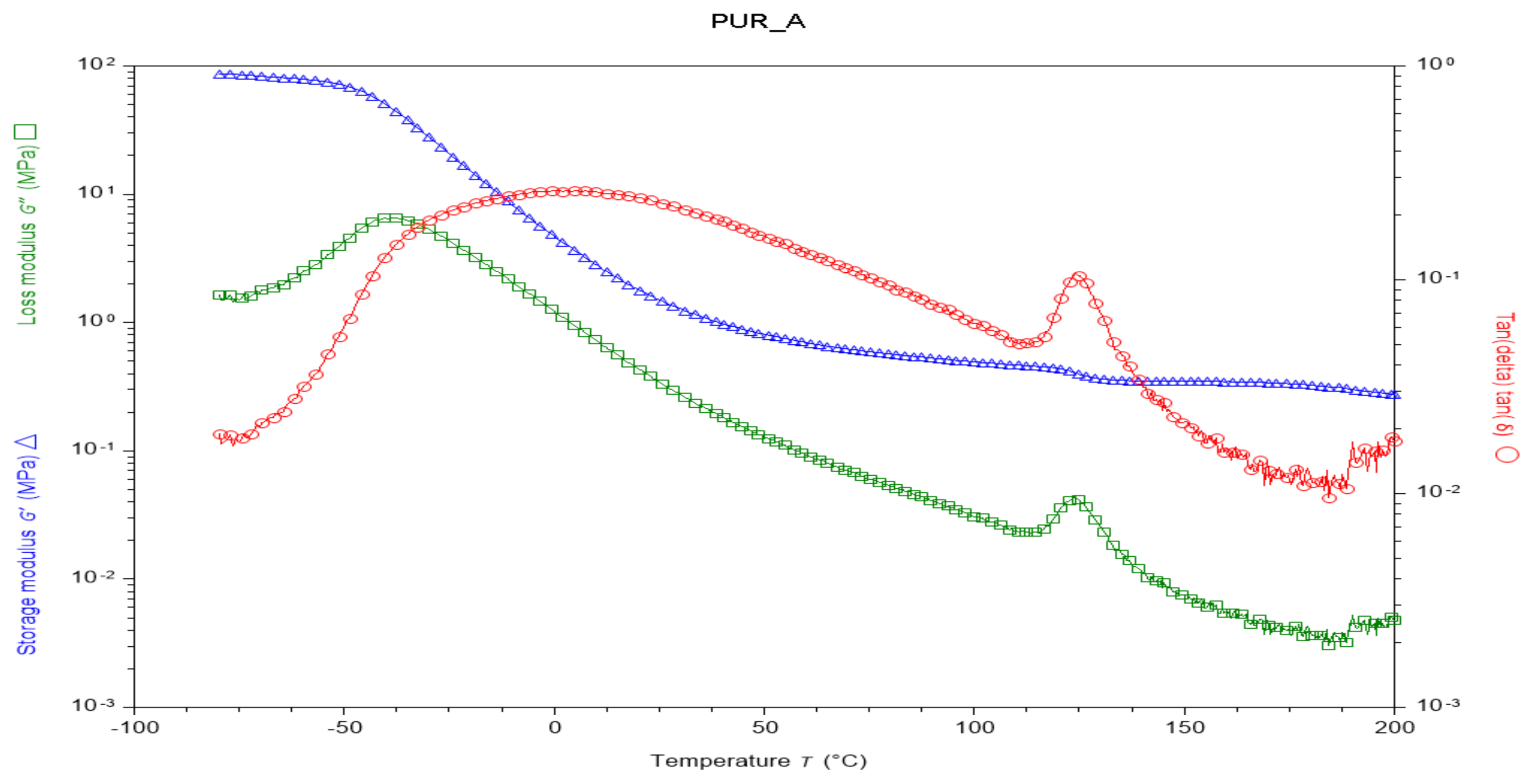

Supplement: Figure S3 — Mechanical dynamic analysis (DMA) results of polyurethane foam. [file turkjchem-46-3-814s3a.tif]

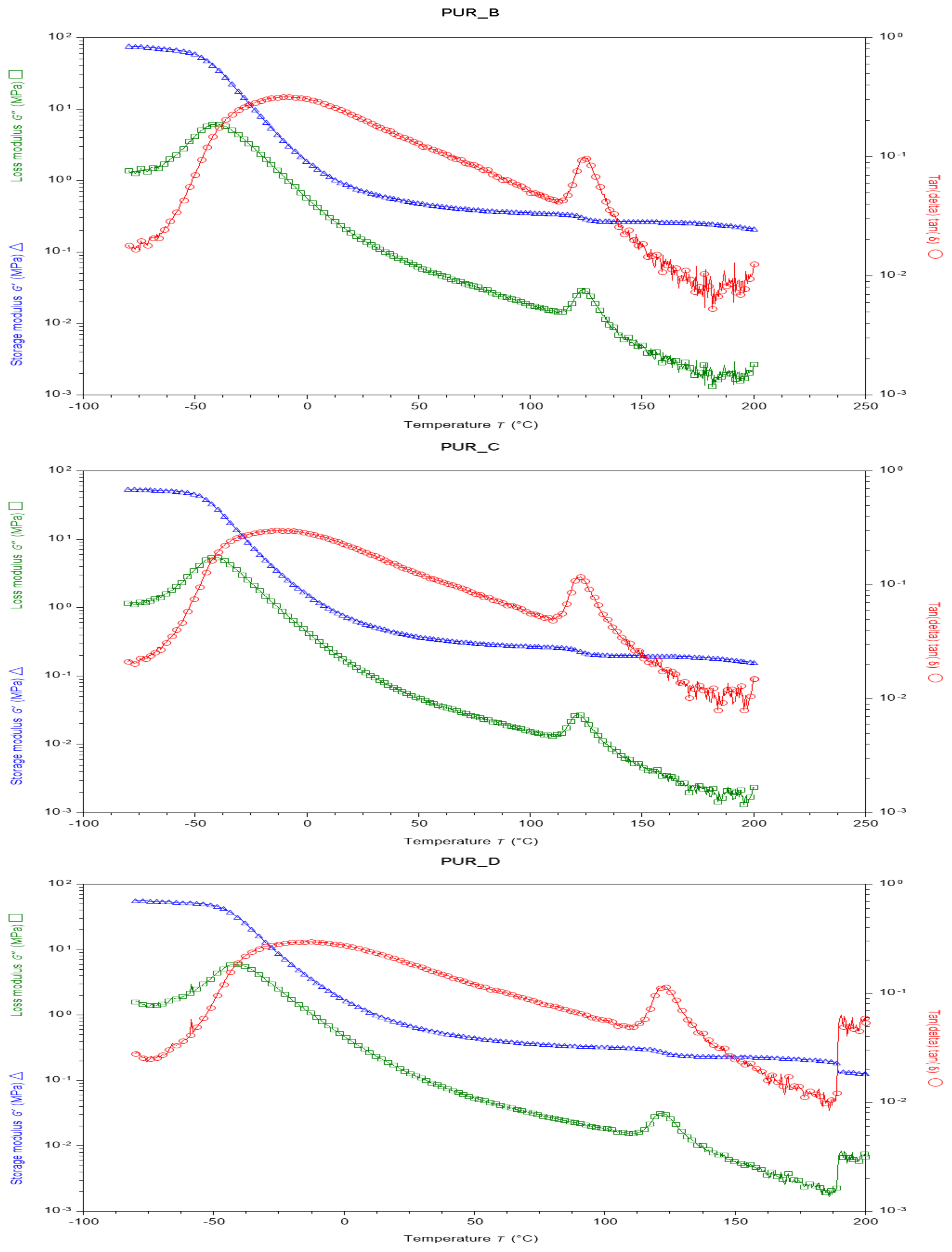

Supplement: Figure S3 — Mechanical dynamic analysis (DMA) results of polyurethane foam. [file turkjchem-46-3-814s3b.tif]

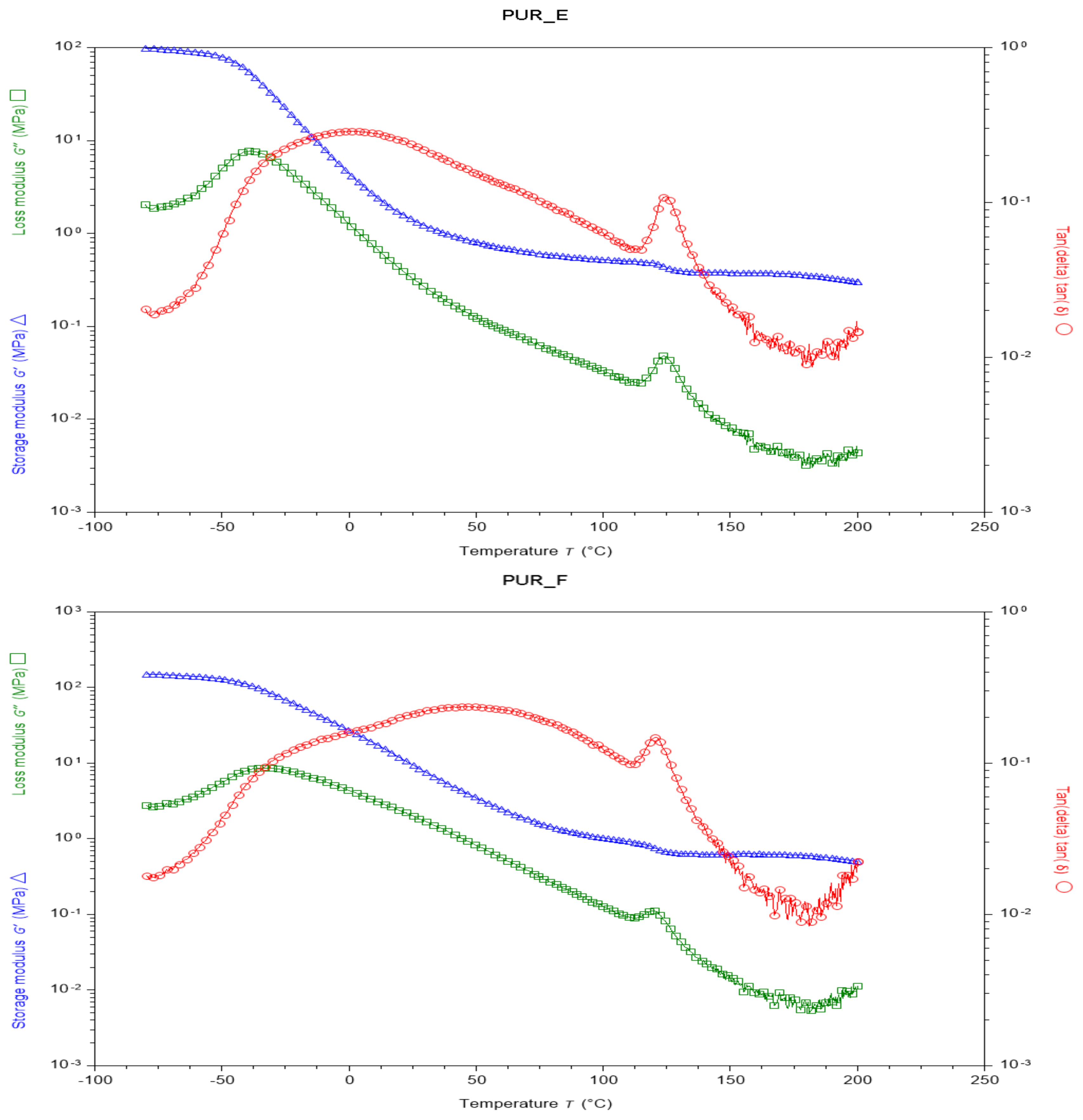

Supplement: Figure S3 — Mechanical dynamic analysis (DMA) results of polyurethane foam. [file turkjchem-46-3-814s3c.tif]
